# Oral administration of PPC enhances antigen-specific CD8^+ ^T cell responses while reducing IgE levels in sensitized mice

**DOI:** 10.1186/1472-6882-9-49

**Published:** 2009-11-30

**Authors:** Mike Burrows, Deepak Assundani, Esteban Celis, Frank Tufaro, Akiko Tanaka, W Guy Bradley

**Affiliations:** 1 Tampa Bay Research Institute, 10900 Roosevelt Blvd. N., St. Petersburg, FL 33716, USA; 2 H. Lee Moffitt Cancer Center & Research Institute, 12902 Magnolia Drive, Tampa, FL 33612, USA; 3 Allera Health Products, Inc. 360 Central Avenue, Suite 1560, St. Petersburg, FL 33701, USA

## Abstract

**Background:**

For almost 2000 years it has been recognized that aqueous extracts from pine cones possess medicinal properties beneficial for the treatment of a broad variety of diseases and conditions. In this report, the ability of an orally administered poly phenylpropanoid-polysaccharide rich extract of pine cones (PPC) to suppress the generation of IgE and to significantly enhance antigen-specific cellular responses to a variety of vaccines was tested.

**Methods:**

A variety of vaccine protocols were utilized to determine the affects of orally administered PPC on the Th1/Th2 cytokine balance, the production of IgE antibodies, and the generation of antigen-specific cytotoxic T cells. The effect of PPC on the Th1/Th2 balance in aged mice was also investigated.

**Results:**

Oral delivery of PPC was found to significantly suppress serum IgE levels in naïve mice and in mice sensitized to ovalbumin. PPC was also found to enhance the generation of antigen-specific CD8^+ ^T cells in mice immunized with DNA, dendritic cell, and soluble protein vaccines. The suppression of IgE was associated with reduction of IL-4 secretion and the enhanced production of IL-12 and IFN_γ _by antigen-stimulated splenocytes from PPC treated mice. PPC also suppressed the Th2 response and enhanced the Th1 response of splenocytes from aged mice.

**Conclusion:**

Oral delivery of PPC enhances the generation of an antigen-specific CD8^+ ^T cell responses induced by soluble protein, DNA, and dendritic cell vaccines while at the same time suppressing the generation of a Th2 dominant IgE response. This effect on the Th1/Th2 balance was also observed in aged mice.

## Background

Almost 2000 years ago the knowledge that aqueous extracts of pine cones possessed medical properties was first recorded [1,2]. For much of the last century the Japanese inhabitants on the island of Kyushu have also known about the medicinal properties of pine cones. They have used a pine cone tea to treat illnesses ranging from colds to cancer [3]. Because of the beliefs that the pine cone tea is able to treat such a broad range of illnesses, it was hypothesized by us that the effects of the extract were likely being mediated through the immune system.

PPC is a poly-phenylpropanoid-polysaccharide complex prepared from a single species of pine cones [4]. Proligna™ is a proprietary powder form of PPC that has been manufactured by Allera Health Products, Inc. (St. Petersburg, FL) and is commercially available as Immune Extra™. The polysaccharide component is composed of mannose, fucose, arabinose, galactose, glucose, and uronic acids [5]. The extraction method used to prepare PPC is highly reproducible as determined by UV spectroscopy, FPLC, and biological assays.

In investigations where the toxicity of PPC has been examined in mice, oral administration at doses as high as 1.83 g/kg produce no noticeable toxic effects [6,7]. However, in contrast to the apparent safety associated with oral administration of the extract, intravenous injection of mice with one-thirtieth the oral dose was found to induce a toxic shock-like syndrome and then death within two hours of administration. The LD_50 _of the intravenously delivered extract was found to be 52.5 and 56.0 mg/kg for male and female mice [8]. Orally effective concentrations of PPC have been determined in a variety of murine vaccine studies and plateau at a dose of 40 mg/kg/day.

Tissue distribution of the orally administered extract (10 mg/kg) was examined by measuring the levels of ^125^iodine-labeled extract in a variety of murine tissues at intervals of 3, 24, or 48 h after delivery [7]. After 3 h approximately 61% of the recoverable radioactivity was found in the stomach and small intestine while 6.6% was recovered from the blood, 4.4% was recovered from the liver, and 9.5% was recovered from the urine. Twenty-four hours after delivery, 89% of the recoverable activity was detected in the urine and feces [7]. These results suggest that while the majority of the extract remains in the GI tract and is excreted within 24 h, a small fraction might be absorbed systemically.

While the pine cone extract has been traditionally used to complement the treatment of a variety of illnesses, an increasing number of people using the commercial product have remarked that when taken on a daily basis it provides significant relief from, or even eliminates, their allergy symptoms. Since numerous studies have demonstrated a strong correlation between the reduction of serum IgE levels and noticeable improvement in the well being of people suffering from IgE-mediated allergies [9-12] we sought to determine if PPC might be modulating the production of IgE.

The results presented herein suggest that PPC might in fact be able to reduce IgE levels in patients with allergy. Oral administration of PPC was found to reduce serum IgE levels in mice and to significantly suppress the development of an allergen induced IgE response. Interestingly, the potential of PPC to suppress IgE levels appears to be associated with its ability to enhance the generation of a Th1 - associated cellular immune response, a response demonstrated to improve the activity of a variety of vaccine types.

## Methods

### Mice

Six week old female C57BL/6 and Balb/c mice were purchased from Charles River Laboratories. Aged male B6C3F1 mice (23-25 months old) were obtained from the National Institute of Aging. The mice were maintained in accord with the NIH *Guide for the Care and Use of Laboratory Animals*. Experimental procedures were performed in accord with research protocols approved by the Tampa Bay Research Institute's Institutional Animal Care and Use Committee (Assurance number: A3062-01). The results presented herein are from single experiments representative of the findings obtained from multiple replicates.

### Preparation of PPC

Pine cones from Scotch pine (*Pinus sylvestris*) were washed extensively in deionized water. The pine cones were then loaded into a stainless steel reaction vessel and extracted with water at elevated pH and high temperature. The resulting extract was standardized by visible and UV spectroscopy and by a series of biological assays. The liquid was tested extensively to ensure that it did not contain contaminants such as herbicides, pesticides, heavy metals, or microorganisms and was then spray dried to form a dark brown, odorless, stable powder (Proligna™). The over the counter form, marketed as Immunextra^®^, consists of 16 mg Proligna™ and 200 mg microcrystalline cellulose in a vegetarian capsule. Product stability and potency are tested by independent laboratories. Allera Health Products, Inc. complies with FDA guidelines for manufacturing dietary supplements in the United States and is a member of the Natural Products Association.

The powdered form of PPC, Proligna™, was obtained from Allera Health Products Inc. (St. Petersburg, FL), suspended in sterile water to a stock concentration of 25 mg/mL (w/v), centrifuged at 10,000 × g for 20 minutes and then filtered through a 0.2 μm nylon filter. The stock solution was diluted in sterile distilled water to prepare as drinking water for the mice.

### Immunizations

Female C57BL/6 mice were injected subcutaneously with either 100 μg of ovalbumin (Grade V, Sigma Chemical Co, St. Louis, MO) or 500 μg ovalbumin along with either 100 μg CpG (ODN 1826) (provided by Mayo Clinic Molecular Core, Rochester MN) or 50 μg poly I:C (Sigma Chemical Co., St. Louis, MO) formulated in IFA on day 0 (priming) and then again on day 14 (boost). In one set of experiments female Balb/c mice were injected i.p. with 10 μg ovalbumin formulated in 2 mg Imject alum (Thermo Fisher Scientific, Rockford, IL) on day 0 (prime) and again on day 14 (boost).

### Cell lines

#### Generation of BM-DC

Hind limbs of C57BL/6 mice were harvested and bone marrow was isolated. Bone marrow derived dendritic cells (BM-DC) were generated by plating bone marrow cells at 1 × 10^6 ^cells/mL in RPMI media (Invitrogen, Carlsbad, CA) supplemented with 10% FCS (Invitrogen) for 7 days with murine GM-CSF (10 ng/mL) and murine IL-4 (5 ng/mL) (Peprotech, Rocky Hill, NJ). Media was replaced periodically and fresh GM-CSF and IL-4 was added to the cells. At day 7, BM-DC were harvested and replated at 2 × 10^6 ^cells/mL with 1 μg/mL of LPS to induce maturation overnight. On day 8, BM-DC were harvested and pulsed with 10 μg/mL of peptide for 2 hours at 37°C and 5% CO_2_.

#### DC Vaccine

C57BL/6 mice (3 per group) were immunized by i.v. injections with 2 × 10^6 ^LPS-matured BM-DC, pulsed with either the MHC-Class I (H-2Kb) restricted OVA_257-264 _(SIINFEKL) or Trp2_180-188 _(SVYDFFVWL) peptides (A&A Labs., San Diego, CA). Each mouse received the DC vaccine injections on days 0, 7, and 14. For some groups, PPC (200 μg/mL) was added in the drinking water, beginning 24 hours before the first vaccination and continued throughout the period of the study.

#### DNA Vaccination

Mice (3 per group) were immunized with a total of 100 μg of plasmid DNA suspended in 100 μL normal saline. A total of 50 μL of the DNA solution was then injected into each of 2 sites in the *quadriceps femoris *muscles. Immunization was followed immediately by electroporation of the injected area (95 volts in four pulses of 65 milliseconds) using an Electro Square Porator device (BTX, model TX830). All groups of mice were vaccinated twice with a 2 week interval. Some groups of mice received 200 μg/mL PPC in their drinking water, starting 24 hours before the first vaccination and continuing throughout the duration of experiment.

#### Detection of serum IgE

Serum from mice was analyzed by ELISA using the antibody kit from Bethyl Laboratories (Montgomery, TX) and following the manufacturer's instructions. The sensitivity of the assay was 3.9 ng/mL for murine IgE. Assays were performed in 96 well flat bottom Maxisorp plates (Nunc; Thermo Fisher Scientitific, Rochester, NY) using serum diluted 1:10 with 50 mM Tris, 0.14 M NaCl, 1% BSA, and 0.05% Tween 20.

#### Detection of OVA-specific IgG_2a_

Maxisorp 96 well flat-bottom plates were coated with 100 μg/mL ovalbumin in carbonate-bicarbonate buffer (Sigma) overnight at 10°C. The plates were washed with PBS containing 0.05% Tween-20 (PBST) and then blocked by adding 300 μL StartingBlock (Pierce Scientific, Rockford, IL) to each well for 10 min. This was removed and 2-fold serial dilutions of the serum samples, ranging from 1:250 to 1:32,000, in PBS containing 1% bovine serum albumin were added in triplicate to appropriate wells and incubated overnight at 10°C. After the overnight incubation the wells were washed 4 times with PBST and then horseradish peroxidase-conjugated goat anti-mouse IgG_2a _antibody (cat no. M32307, Invitrogen, Carlsbad, CA), diluted 1:2000, was added to each well for 1 h at room temperature. The wells were washed with PBST and then 100 μl of the SureBlue substrate (KPL, Inc., Gaithersburg, MD) was added. The color reaction was stopped by adding 50 μL of 2 M H_2_SO_4 _or 1 M HCl and the absorbance was read at 450 nm.

#### Splenocyte isolation

To prepare single cell suspensions of splenocytes the spleens were isolated using aseptic technique, placed in sterile polypropylene bags containing 5 mL of incomplete media (RPMI 1640), and homogenized in a Stomacher 80 Laboratory Blender (Seward Medical, London, England) at high setting for 60 seconds. The resulting cell suspension was filtered through a 70 μm nylon and then mixed with 5 mL of 2× ACK lysis buffer (1× ACK lysis buffer is 8.29 g/L NH_4_Cl, 1 g/L KHCO_3_, and 37.2 mg/L Na_2_EDTA, pH 7.2) for 5 minutes at room temperature to remove the RBC. The splenocytes were pelleted and then suspended in complete media (RPMI 1640 containing 10% fetal calf serum, 4 mM L-glutamine, and 50 μM β-2 mercaptoethanol) to a final concentration of 1 × 10^6 ^per mL. The cell count and viability was performed using the trypan blue dye exclusion method. The viability of the cells was typically >95%.

#### Splenocyte cultures for cytokine production

One milliliter of splenocytes, at a concentration of 1 × 10^6 ^cells/mL (from aged B6C3F1 mice) was plated in each of six wells of a 24 well plate. To enhance cytokine production in the splenocyte culture, 50 μL of a 100 μg/mL solution of concanavalin A (ConA) was added to 3 of the 6 wells. After 48 hours of incubation at 37°C in an atmosphere containing 5% CO_2_, the culture media was removed and placed in a sterile 1.5 mL microcentrifuge. The media was cleared of cells and debris by centrifugation at 16,000 × g for 2 minutes. The cleared media was transferred to a sterile 1.5 microfuge tube and stored at -80°C until it was analyzed by ELISA for cytokine levels.

Splenocytes from Balb/c mice that had been immunized in the presence or absence of PPC were incubated in 24 well plates at a concentration of 4 × 10^6^/mL for 48 h in the presence of media alone, 100 μg/mL ovalbumin (Fraction V, Sigma Chemical Company), or 10 ng/mL PMA + 100 ng/mL ionomycin. The 48 h supernatants were cleared of cells and then analyzed by ELISA for the presence of appropriate cytokines.

#### IFNγ ELISPOT

For detection of CD8^+ ^T cells secreting IFNγ, enzyme linked immunosorbent spot (ELISPOT) assays were performed. Briefly, Millipore Multiscreen Plates were coated by overnight incubation at 4-10°C with 100 μl of 5 μg/mL anti-mouse IFNγ (clone AN-18, eBioscience, Inc., San Diego, CA) in PBS and then blocked by incubation for 3 h at 37°C with 200 μL RPMI-10 (RPMI 1640 with 10% fetal bovine serum). Stimulator cells were washed once with complete media and then suspended at a concentration of 3-5 × 10^6^/mL. The appropriate peptide was then added at a concentration of 20 μg/mL and the cells were incubated for a minimum of 3 hours in a 5% CO_2 _atmosphere at 37°C. The stimulator cells were then irradiated at 10,000-20,000 Rads, depending on the cell line being used. The stimulator cells were washed twice in RPMI-10 and then suspended in RPMI-10 to a concentration of 1 × 10^6^/mL. For the IFNγ ELISPOT assay, irradiated stimulator cells (EL4 or EG7) were used to present endogenous OVA or were pulsed with the Class I OVA peptide (SIINFEKL).

CD8^+ ^T cells were purified from splenocytes of vaccinated mice by positive selection using antibody-coated magnetic beads (Miltenyi Biotec, Auburn, CA). Responder (CD8 purified) cells were incubated at different concentrations per well, together with 5 × 10^4 ^stimulator cells (peptide pulsed, unpulsed, or expressing tumor cells). Cultures were incubated at 37°C for 20 h. After the incubation the cells were removed from the plate and the wells washed 6 times with PBST. Then, 100 μL of biotinylated anti-IFNγ at 1 μg/mL in PBST was added to each well, the plate was covered and incubated 2-3 hours at room temperature. The plate was then washed with PBST and 100 μL/well of avidin-HRP was added at a 1:1000 dilution in PBST followed by incubated at room temperature for 1 hour. The plate was washed 6 times with PBST and then 3 times with PBS only. Freshly prepared 3-amino-9-ethylcarbazole (AEC) substrate (100 μL) was added to each well and incubated for 10-30 minutes at room temperature. The plate was flooded with tap water to stop the color development and then submerged in a tank of tap water to assure low background. The plates were allowed to dry overnight. Spot counting was performed with an AID EliSpot Reader System (Autoimmune Diagnostika GmbH).

#### Cytokine ELISA

The detection of the cytokines, IL-12p40, IFNγ, IL-4, and IL-10 was performed using the murine cytokine development kits from Peprotech (Rocky Hill, NJ). The IL-5 ELISA was performed using the Ready-SET-Go kit from eBioscience (San Diego, CA). The assays were performed following the manufacturer's instructions. The sensitivities for the IL-12p40, IFNγ, IL-4, IL-5 and IL-10 ELISAs were, 32, 16, 16, 4, and 47 pg/mL, respectively.

### Statistical Analysis

The results are expressed as means ± SD (standard deviation). All assays were perform in triplicate and repeated at least three times. The statistical difference between two groups was determined by Students t test. When multiple groups were being analyzed the statistical difference was determined using the one-way analysis of variance (ANOVA). A *p*-value < 0.05 was considered statistically significant.

## Results

### Oral administration of PPC reduces total IgE levels in naïve mice

**Figure 1:**
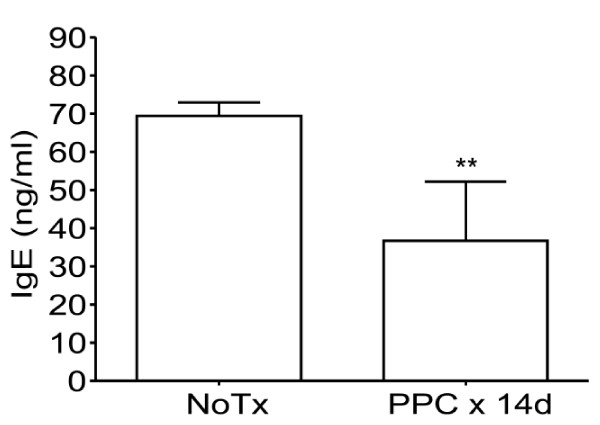
**Reduction of basal IgE in naïve mice**. Naïve Balb/c mice (n = 4/group) were provided water or 200 μg/mL PPC *ad libitum *for 14 days. The serum levels of total IgE were measured by ELISA. PPC suppressed the development of IgE (** represents a *p *< 0.01). Error bars represent the standard deviation.

To determine if oral administration of PPC affected IgE levels in mice, we administered a group of naive female Balb/c mice (n = 4 per group) with 200 μg/mL of PPC in their drinking water *ad libitum *for 14 days, after which, the serum levels of total IgE were determined by ELISA. The IgE levels in mice receiving PPC were found to be significantly lower (*p *< 0.01) that those found in mice receiving just water (Figure 1).

### Continuous delivery of PPC suppresses the rise in serum IgE levels associated with sensitization to an allergen

**Figure 2:**
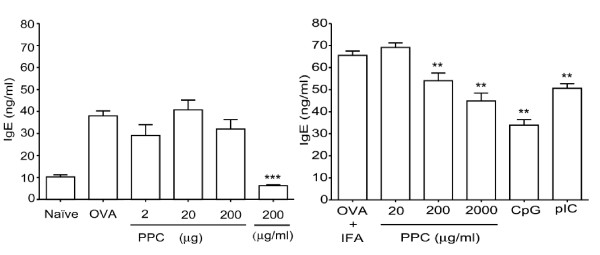
**Suppressed induction of an IgE response**. Mice were injected s.c. on day 0 and day 14 with 100 μg OVA (left panel) or 500 μg OVA formulated in IFA (right panel). CpG (100 μg s.c.) or poly I:C (50 μg s.c.) were delivered at the time of priming and booster injections to serve as positive controls. Mice in the left panel received 2, 20, or 200 μg PPC by gavage at the time of the priming and booster injections, or 200 μg/mL PPC in the drinking water *ad libitum*, beginning at the time of the priming injection. Mice in the right panel were provided PPC (20, 200, or 2000 μg/mL) *ad libitum *in their drinking water beginning on day -1 and lasting until day 21 when the total serum IgE ELISA assays were performed. All mice receiving PPC also received the OVA vaccine. Error bars represent the standard deviation. The (**) represents a *p *< 0.001 and (***) represents a *p *< 0.0001.

C57BL/6 mice (4 per group) were injected s.c. on days 0 and 14 with 100 μg chicken egg ovalbumin (OVA) (Figure 2, left panel) or 500 μg of OVA formulated in IFA (Figure 2, right panel) to determine if oral delivery of PPC could suppress the induction of an IgE response. The TLR ligands, CpG (100 μg) and poly I:C (50 μg), were formulated with the OVA and IFA and provided with both the priming and booster injections to serve as positive controls for IgE inhibition. In the first experiment, groups of mice received either 2, 20, or 200 μg of PPC by gavage at the time of the priming and booster injections, or 200 μg/mL PPC in the drinking water *ad libitum*, beginning immediately after the priming injection (Figure 2, left panel). In the second set of experiments, groups of mice were provided PPC (20, 200, or 2000 μg/mL) *ad libitum *in their drinking water beginning on day -1 and lasting until day 21, when the total serum IgE ELISA assays were performed (Figure 2, right panel). The results demonstrate that delivery of PPC by gavage at the time of the initial sensitization and then at the time of the booster inoculation had no significant effect on the levels of serum IgE (p > 0.05 at all three doses of PPC, left panel). However, when PPC was provided *ad libitum *at 200 μg/mL, a highly significant suppression of the allergen-associated increase in total IgE levels was detected (Figure 2, left panel, *p *< 0.0001).

When the C57BL/6 mice were immunized with 500 μg OVA formulated in IFA (Figure 2 right panel), a mixture that biases a Th2 mediated response [13], PPC provided *ad libitum *demonstrated no effect at 20 μg/mL (p > 0.05), but was able to significantly suppress the IgE response at 200 and 2000 μg/mL (*p *< 0.001 at both doses). As anticipated, both of the positive controls, CpG and poly IC were found to suppress the allergen-associated rise in IgE levels (*p *< 0.001 for both, Figure 2, right panel).

### Continuous delivery of PPC enhances the production of allergen-specific IgG_2a_

**Figure 3:**
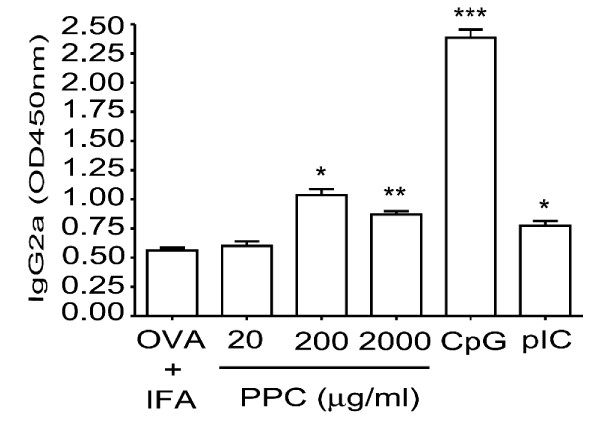
**Enhancement of OVA-specific IgG_2a _production**. C57BL/6 mice (4/group) were injected s.c. on day 0 and day 14 with 500 μg OVA formulated in IFA. CPG (100 μg s.c.) or poly I:C (50 μg s.c.) were delivered at the time of priming and booster injections to serve as positive controls. Mice were provided PPC (20, 200, or 2000 μg/mL) *ad libitum *in their drinking water beginning on day -1 and lasting until day 21 when the OVA-specific IgG_2a _ELISA assays were performed. Error bars represent the standard deviation. The (*) represents a *p *< 0.05, the (**) represents a *p *< 0.01, and (***) represents a *p *< 0.001.

Since it appeared that PPC was capable of suppressing an IgE response, a response known to be highly dependent on the Th2-associated cytokine, IL-4, we sought to determine if PPC might be suppressing the Th2 response by inducing the mutually inhibitory Th1 response. To do this we measured the serum levels of OVA-specific IgG_2a_, an isotype strongly associated with the presence of a Th1 response. Sera obtained from the C57BL/6 mice immunized with OVA + IFA were examined by ELISA (Figure 3). When the mice were immunized with 500 μg OVA formulated in IFA, PPC provided *ad libitum *at 20 μg/mL failed to enhance the IgG_2a _response (p > 0.05). However, when the mice received either 200 or 2000 μg/mL along with vaccination, a significant increase in the production of OVA-specific IgG_2a _antibodies was detected (*p *< 0.05 and <0.01, respectively, Figure 3). The finding that the same doses of PPC that suppress IgE production also enhanced OVA-specific IgG_2a _levels, suggest that PPC alters the Th1/Th2 balance. As expected, both of the TLR ligands were found to enhance production of the OVA-specific IgG_2a _(*p *< 0.001 for CpG and < 0.05 for poly IC).

### Continuous delivery of PPC enhances an antigen-specific CD8^+^/IFNγ^+ ^T cell response

**Figure 4:**
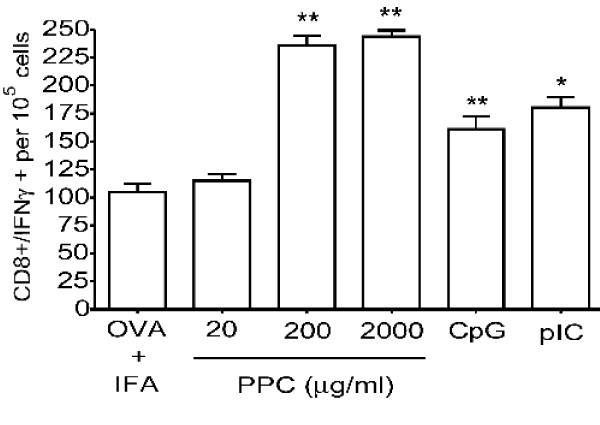
**PPC enhances the production of IFNγ^+^/CD8^+ ^T cells in response to a protein vaccine**. C57BL/6 mice (4/group) were injected s.c. on day 0 and day 14 with 500 μg OVA formulated in IFA. CPG (100 μg) or poly I:C (50 μg) was included in the priming and booster injections to serve as positive controls. Other mice were provided PPC (20, 200, or 2000 μg/mL) in their drinking water beginning on day 7 and lasting until day 21 when the IFNγ ELISPOT analysis was performed. The number of spots (CD8^+/^IFNγ^+ ^cells per 10^5 ^isolated CD8^+ ^cells) is shown. Error bars represent the standard deviation. The (*) represents a *p *< 0.006, while the (**) represents a *p *< 0.001.

To determine if the observed suppression of IgE and enhancement of allergen-specific IgG_2a _were associated with overall enhancement of a Th1-type response, we determined whether PPC affected development of an OVA-specific CD8^+ ^T cell response. Splenic CD8^+ ^T cells were isolated on day 21 from C57BL/6 following immunization with 500 μg OVA formulated in IFA. The TLR ligands, CpG (100 μg) and poly IC (50 μg) were included in the formulation to serve as positive controls for immune modulators. Mice in three other groups (n = 4 per group) were provided PPC (20, 200, or 2000 μg/mL) in their drinking water beginning on day -1 and lasting until day 21 when an IFNγ ELISPOT analysis was performed (Figure 4). Both the CpG and poly I:C significantly enhanced the CD8 response against OVA (*p *= 0.001 and 0.006, respectively), while the delivery of PPC at 200 and 2000 μg/mL more than doubled the response (Figure 4, *p *< 0.001 for both doses). As was observed for its effects on IgE and IgG_2a_, PPC provided at 20 μg/mL did not significantly affect the CD8^+ ^T cell response (*p *> 0.05). When compared to CpG and poly I:C, the oral delivery of PPC at 200 and 2000 μg/mL was more effective at enhancing the OVA-specific CD8^+ ^T cell response (Figure 4).

Combined with the finding that PPC suppresses IgE levels in mice, and that in mice this has been associated with elevation of antigen-specific IgG_2a _and a doubling of the number of antigen-specific CD8^+^/IFNγ T cells, these results suggest that oral administration of PPC is capable of significantly boosting a Th1-like response and that this response, via the mutual inhibitory feedback mechanism, could be responsible for suppressing the generation of a Th2 response and the production of IgE.

### PPC suppresses Th2-associated cytokine production by splenocytes from Balb/c mice sensitized to ovalbumin

**Figure 5:**
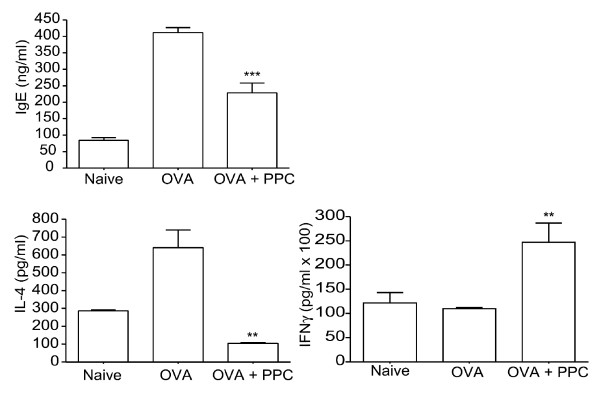
**PPC affects the Th1/Th2 cytokine expression pattern**. Balb/c mice (4/group) were injected i.p. on day 0 and day 14 with 10 μg OVA formulated in alum and then on day 28 the serum IgE levels were measured. Mice in the OVA + PPC group received 200 μg/mL of PPC in their drinking water beginning 7 days before (day -7) the first OVA injection and continued throughout the entire 28 day period. The serum IgE levels are shown for untreated (Naïve), OVA vaccinated (OVA) and PPC treated mice that had been vaccinated with OVA (OVA + PPC). The differences between the IgE and IL-4, and IFNγ levels in the OVA and OVA + PPC groups are significant (*p *< 0.001 and 0.01, 0.01, respectively). Error bars represent the standard deviation. The (**) represents a *p *< 0.01 while the (***) represents a *p *< 0.001.

To demonstrate that PPC could be affecting the Th1/Th2 balance, we examined cytokine production by female Balb/c mice exposed to OVA. OVA (10 μg) formulated in alum was injected i.p. on days 0 and 14. One group of mice (OVA + PPC) received 200 μg/mL PPC in their drinking water beginning 7 days prior to the first injection. On day 28, fourteen days after the last OVA injection, the serum levels of IgE were determined and the *in vitro *cytokine production by OVA stimulated splenocytes was examined (Figure 5). The results reveal that in this Th2 dominant mouse, oral administration of PPC significantly suppressed the generation of IgE in response to the immunization (*p *< 0.001) and suppressed the production of IL-4 in response to OVA (*p *< 0.01). PPC also enhanced the release of IFNγ significantly (*p *< 0.01) (Figure 5).

### PPC enhances IL-12 and suppresses IL-4 and IL-10 secretion by splenocytes from aged mice

**Figure 6:**
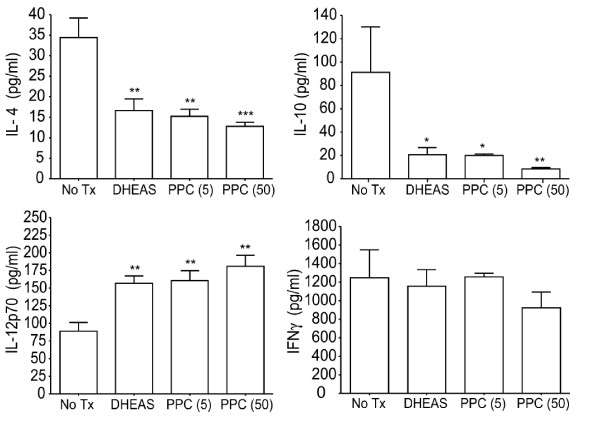
**PPC affects the Th1/Th2 pattern of cytokine expression in aged mice**. Aged B6C3F1 mice were provided PPC (5 or 50 μg/mL) or DHEAS (100 μg/mL) *ad libitum *for 25 days. The splenocytes from these mice were then cultured in the presence of 5 μg/mL concanavalin A for 48 h. The culture supernatant was examined by ELISA for the levels of IL-4, IL-10, IL-12, and IFNγ. Error bars represent the standard deviation. The (*) represents a *p *< 0.05, the (**) represents a *p *< 0.01, and the (***) represents a *p *< 0.001.

To determine if PPC could affect the Th1/Th2 balance in a very different immunologic model, we administered aged B6C3F1 mice (23-25 month old males) with relatively low levels of PPC (5 or 50 μg/mL) in their drinking water *ad libitum *for 25 days. One group of mice received DHEAS (dehydroepiandrosterone sulfate, 100 μg/mL) in their drinking water as a positive control. DHEA serves as the precursor to testosterone and estrogen and has been reported to enhance the Th1 response in aged mice and humans [14]. Each treatment group contained a total of 15 mice. After 25 days, 5 mice per group were randomly selected and euthanized. The spleens were isolated and converted to single cell suspensions, cultured in the presence of 5 μg/mL concanavalin A for 48 h, and then the culture supernatant was examined for the levels of Th1 and Th2 cytokines (Figure 6). The results demonstrate that the splenocytes from PPC treated mice produced significantly less IL-4 (*p *< 0.01 for 5 μg/mL and < 0.001 for 50 μg/mL) and IL-10 (*p *< 0.05 for 5 μg/mL and <0.01 for 50 μg/mL), and significantly more IL-12p70 (*p *< 0.01 for both doses) than splenocytes from untreated mice. At the low levels provided, PPC did not appear to significantly affect the amount of IFNγ in these mice.

These results demonstrate that the oral delivery of PPC can modulate an immune response away from one that is predominately Th2-mediated towards one that is Th1-mediated. If this were the case, we would expect to find that oral delivery of PPC would enhance the antigen-specific CD8^+ ^T cell response to a variety of vaccine types and suppress the development of IgE antibodies.

### PPC enhances the CD8^+^/IFNγ^+ ^T cell response to a OVA-specific DNA vaccine

Female C57BL/6 mice (4 per group) were immunized by electroporation with plasmid DNA encoding full length ovalbumin and then analyzed for the generation of IFNγ CD8^+ ^T cells specific for the dominant Class I epitope (OVA_257_, SIINFEKEL).

**Figure 7:**
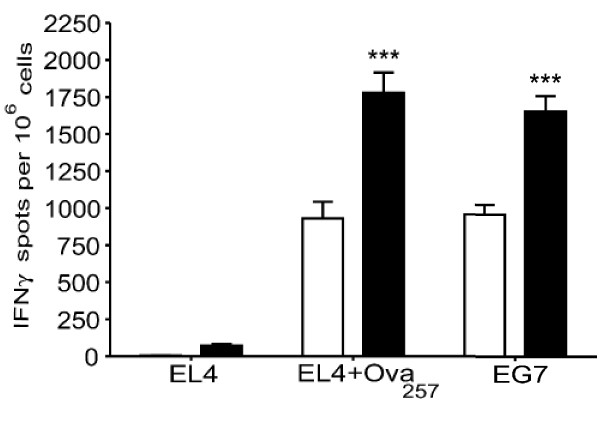
**PPC enhanced the IFNγ^+^/CD8^+ ^T cell response to a DNA vaccine specific for ovalbumin**. PPC enhanced the DNA vaccine induced production of OVA-specific IFNγ^+^/CD8^+ ^T cells. Mice were immunized by DNA injection followed immediately by electroporation. Mice were injected with DNA encoding full-length ovalbumin and then the injection area was immediately exposed to 95 volts in four pulses of 65 milliseconds using an Electro Square Porator device (BTX, model TX830). CD8^+ ^T cells were isolated from the splenocytes population and plated with stimulator cells (EL4 only, EL4 pulsed with the OVA_257 _peptide, or EG7 cells). Mice receiving no PPC are represented by the open bars while those receiving 200 μg/mL PPC *ad libitum *are represented by the filled bars. Error bars represent the standard deviation. The delivery of PPC was found to significantly enhance the number OVA specific IFNγ^+^/CD8^+ ^T cells (*p *= 0.009 for EL4 OVA_257 _and *p *= 0.005 for EG7).

While untreated mice produced 800 IFNγ spots per 10^6 ^isolated CD8^+ ^T cells, co-administration of 200 μg/mL of PPC more than doubled that number (1750). This effect was significant (*p *= 0.009 for OVA_257 _peptide pulsed EL4 and 0.005 for the cells stimulated with EG7 cell) and antigen-specific since the EL4 without peptide failed to stimulate IFNγ production (Figure 7).

### PPC enhances the CD8^+^/IFNγ^+ ^T cell response to a melanoma antigen-specific dendritic cell vaccine

**Figure 8:**
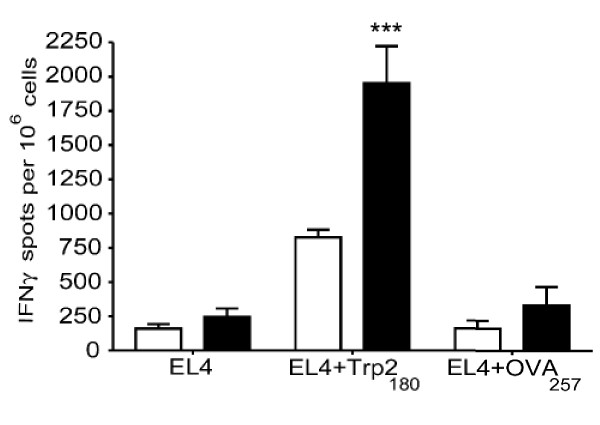
**PPC enhanced the IFNγ^+^/CD8^+ ^T cell response to a dendritic cell vaccine specific for a melanoma antigen**. PPC enhanced the dendritic cell vaccine induced production of melanoma associated Trp2-specific IFNγ^+^/CD8^+ ^T cells. CD8^+ ^T cells were isolated from the splenocytes population and plated with stimulator cells (EL4 only, EL4 pulsed with the Trp2_180 _peptide, or EL4 pulsed with the irrelevant OVA_257 _peptide). Mice receiving no PPC are represented by the open bars while those receiving 200 μg/mL PPC *ad libitum *are represented by the filled bars. Error bars represent the standard deviation. The delivery of PPC was found to significantly enhance the number Trp2_180 _specific IFNγ^+^/CD8^+ ^T cells (*p *= 0.002 for EL4 Trp2_180_).

Female C57BL/6 mice (4 per group) were immunized weekly for three weeks by intravenous injection of 2 × 10^6 ^LPS-matured bone marrow derived dendritic cells that had been pulsed with the melanoma-derived Trp2_180-188 _peptide (SVYDFFVWL). For some groups, PPC (200 μg/mL) was added in the drinking water beginning 24 hours before vaccination and continued throughout the period of the study. Seven days following the last injection, CD8^+ ^T cells were isolated from the spleen and analyzed via ELISPOT for the antigen-specific production of IFNγ. As shown in Figure 8, mice treated with PPC produced more than double the number of antigen-specific CD8^+^/IFNγ^+ ^T cells (*p *= 0.002 for EL4 pulsed with Trp2_180_). The antigen-specific nature of the response was clearly detected by the lack of a response in the absence of the Trp2_180-188 _peptide or in the presence of the irrelevant OVA_257-264 _peptide (Figure 8, EL4 only and EL4 OVA, *p *= 0.1 for both groups).

## Discussion

For centuries people have utilized a tea prepared from pine cones to provide relief from a variety of ailments, including cancer [3]. A description of the medicinal properties of an orally administered pine cone extract can even be found in the *De Materia Medica *of Dioscorides, a five book compilation on the preparation, properties, and testing of drugs, written in A.D. 65, that was the basis for pharmaceutical and herbal writing until the sixteenth century [1,2].

Upon learning that consumers of the commercial pine cone extract (Immune Extra™) had experienced a significant reduction of their allergy symptoms we sought to determine if the product affected the generation of IgE in mice. It was observed that oral delivery of PPC was able to reduce the basal levels of IgE in naive mice Balb/c mice (Figure 1), a strain known to be Th2-biased and to produce elevated levels of IgE. When we stimulated a Th2 response in C57BL/6 mice by immunizing them with an optimal amount of ovalbumin formulated in IFA, we observed that in the presence of PPC the rise in IgE levels was significantly suppressed (Figure 2). Interestingly, the dose response pattern of IgE suppression observed in the immunized mice was reflected in the ability of PPC at 200 and 2000 μg/mL, but not 20 μg/mL, to significantly increase the production of OVA-specific IgG_2a _(Figure 3) and the production of OVA-specific IFNγ/CD8^+ ^T cells (Figure 4). This biasing of the antigen-specific response from one that was Th2-dominant (increased IgE) in the untreated mice to one that was Th1-dominant (suppressed IgE and an increased IgG_2a _and CD8 response) in the PPC treated mice suggested that PPC is likely to be beneficial in disease processes where it would be advantageous to either enhance Th1 or suppress Th2 responses. To determine if the administration of PPC could be in fact beneficial in instances where Th1 enhancement or Th2 suppression were appropriate, we tested its ability to affect the Th1/Th2 balance in the aged and in a variety of vaccine models.

It has been recognized that as the human population ages the incidence of disease increases and the ability of our immune systems to protect us from infectious diseases and cancer, or to respond to vaccines wanes [15]. At the same time, the incidence of autoimmune diseases and allergies tends to increase [16-21]. These observations, and the detection of increased levels of the key Th2 cytokine, IL-4, in aged humans and mice, demonstrates that in the aged there is a shift in the Th1/Th2 balance towards responses that tends to be Th2 dominant. Unfortunately, Th2 dominant responses are not very effective at protecting us against infections and cancer, and in some cases of autoimmunity, can even be pathologic.

To determine if PPC can influence the Th1/Th2 balance in the aged, we provided aged mice with PPC in their drinking water for 25 days and then activated their splenocytes *in vitro*. The splenocytes from the PPC treated mice were found to produce significantly lower levels of IL-4 and IL-10 and higher levels of IL-12p70 that those from the non-treated mice (Figure 7). These results indicated that the Th1/Th2 balance in the aged could be affected by oral administration of PPC. Interestingly, even though IL-12p70 levels were elevated in ConA-stimulated splenocytes cultures from the PPC- and DHEAS-treated mice, we failed to detect a significant enhancement of IFNγ in these cultures. The finding that splenocytes from the DHEAS treated mice also failed to induce IFNγ in the presence of elevated IL-12p70 levels suggests that the lack of IFNγ response was not specifically associated with PPC. In a parallel experiment (not shown), young B6C3F1 mice were also treated with DHEAS and PPC. The ConA-stimulated splenocyte cultures from these mice were found to produce significantly more IFNγ than the cultures from the untreated mice. Also, the levels of IFNγ in the cultures from the young naïve mice were much higher than that found in the cultures prepared from the older naïve mice. This suggests that some age-related phenomenon was preventing the splenocytes from secreting IFNγ. Based on these findings, further experiments will be performed to determine if the suppression of IL-4 and IL-10, concurrent with the enhancement of IL-12p70 production in the aged mice translates into an increased cellular response to vaccines, resistance to infections, and reduction of IgE. The findings from such experiments could be significantly beneficial to the aging population of humans.

To demonstrate the biological relevance of PPC's ability to enhance a Th1 response in younger mice, we examined its influence on the development of antigen-specific CD8^+ ^T cell responses induced by DNA and dendritic cell vaccines. In both vaccine models it was discovered that oral delivery of PPC more than doubled the number of antigen-specific IFNγ^+^/CD8^+ ^T cells generated by the vaccine (Figures 7 and 8). The doubling of the antigen-specific CD8^+ ^T cell response was also observed in PPC treated mice immunized with a soluble whole protein vaccine (Figure 4). These results strongly suggest that orally administered PPC can function as a potent adjuvant for protein, DNA, and dendritic cell vaccines encoding exogenous antigens. In these experiments CpG was included as a positive control and was found to suppress IgE production while boosting the IgG_2a _response to an extent greater than PPC. However, when the effects of PPC and CpG on the production of antigen-specific CD8^+^/IFNγ^+ ^T cells was examined, PPC treated mice appeared to produce greater numbers of these cells. Since PPC is a crude extract likely to contain hundreds or thousands of different molecules, the finding that it had a weaker effect on IgE suppression and IgG_2a _enhancement than CpG, while having a greater effect on the production of CD8^+^/IFNγ^+ ^T cells, is not that surprising. The mixture of molecules in the crude product could certainly result in synergistic, additive, and even counteracting activities that ultimately produce an *in vivo *response similar and different than purified immune modulators. Further experiments need to be performed in order to isolate the various activities in PPC and to determine the efficacy of using this product to enhance the cellular response to a variety of vaccines targeting infectious agents and cancer.

Our results have provided significant evidence that prolonged oral administration of PPC leads to the reduction of serum IgE levels and the enhancement of cellular immune responses. To determine how this might take place we must consider the mechanisms that lead to IgE production. The first step consists of signals that favor the differentiation of naive Th0 cells to a Th2 phenotype, while the second step comprises the action of cytokines and co-stimulatory signals from Th2 cells that stimulate B cells to switch to the production of IgE antibodies. The differentiation of naive Th0 CD4 T cells is determined by the cytokines they are exposed to before and during this response, and by the intrinsic properties of the antigen, antigen dose, and route of presentation. Naïve CD4 T cells being primed by their first encounter with antigen presenting cells (APC) secreting IL-4 tend to develop into Th2 cells, while those encountering APC secreting IL-12 tend to develop into Th1 cells. Interestingly, development of the Th1 and Th2 phenotypes tend to be mutually exclusive [22,23]. The presence of IL-10 and IL-4 suppresses development of Th1 cells, while the presence of IL-12 and IFNγ suppresses the development of Th2 cells. Even established responses can be shifted in the presence of the right cytokine environment. For example, the presence of IL-4 inhibits the development of Th1 cells and shifts Th1 responses to a less-polarized phenotype [24-26].

It is well recognized that in the presence of IL-4 and co-stimulatory signals from Th2 cells, B lymphocytes are stimulated to switch to the production of IgE antibodies, while in the presence of IFNγ, the switch to the production of IgE is suppressed [27]. The crucial role for these cytokines *in vivo *has been demonstrated in mice, where neutralizing antibodies to IL-4, or administration of IFNγ, both lead to an inhibition of IgE responses [28]. Also, transgenic mice homozygous for a mutation that inactivates the IL-4 gene cannot produce IgE. In humans with hyper-IgE syndrome, the delivery of either IFNγ or IFNγ leads to a reduction in serum IgE levels [29].

Based on the critical nature of IL-4 and IFNγ for the induction and suppression of IgE, respectively, we hypothesize that orally administered PPC is inducing the production of IFNγ which in turn suppresses IL-4 production and consequently IgE production. We believe that PPC's induction of IFNγ production is associated with activation of the Th1 pathway by macrophage and/or dendritic cells stimulated by PPC to secrete IL-12 and other pro-Th1 factors. This release of IL-12 and the interaction with Th0 CD4 cells during antigen encounter could lead to the generation of Th1 CD4 T cells, activation of NK cells, and the generation of antigen-specific CD8 effector cells, all of which can then release IFNγ and block the release and the effects of IL-4. Our finding of enhanced IL-12 and IFNγ and reduced IL-4 release by PPC treated mice (Figures 5 and 6) support this hypothesis. Further experiments will be performed to identify the primary cellular targets of PPC and to determine the mechanisms by which oral administration of PPC leads to biologically significant modulation of the Th1/Th2 balance.

## Conclusion

Oral delivery of PPC enhances the generation of an antigen-specific CD8^+ ^T cell responses induced by soluble protein, DNA, and dendritic cell vaccines while at the same time suppressing the generation of a Th2 dominant IgE response. This effect on the Th1/Th2 balance was also observed in aged mice.

## Competing interests

The authors, DA, and EC declare that they have no competing interests. Authors AT and WGB are listed as inventors on patents related to production of PPC and hold stock in the company (Allera Health Products, Inc) selling the commercial product, Immune Extra™. Author MB was employed by Allera during part of the time spent on this research. Author FT is CEO of Allera Health Products, Inc.

## Authors' contributions

Authors EC, AT, FT, and WGB designed the experiments and interpreted the experimental results. WGB and MB performed all animal work and ELISA assays associated with several of the ovalbumin vaccine studies, and the aged mice studies. DA performed work associated with one of the ovalbumin vaccine studies and ELISPOTS and the DNA and dendritic cell vaccine studies. All authors contributed to the manuscript preparation and approved its submission.

## Pre-publication history

The pre-publication history for this paper can be accessed here:

http://www.biomedcentral.com/1472-6882/9/49/prepub
